# Mental Health of Parents and Their Children: A Longitudinal Study of the Effects of Parents’ Negative Affect on Adolescents’ Pathological Gaming

**DOI:** 10.3390/healthcare10112233

**Published:** 2022-11-08

**Authors:** Mei Ying Piao, Eui Jun Jeong, Jeong Ae Kim

**Affiliations:** 1 Department of Digital Culture & Contents, Konkuk University, Seoul 05029, Korea; 2 Department of Humanities Counseling & Therapy, Konkuk University, Seoul 05029, Korea

**Keywords:** pathological gaming, depression, anxiety, mental health, adolescent, parents

## Abstract

Parents’ negative affect could lead to the deterioration of the mental state of their adolescent children. According to previous studies, children of parents who have symptoms of depression or anxiety are more likely to have mental problems. As one of the most important issues concerning adolescents with the rapid rise of screen time, the concern for pathological gaming among adolescents continues to intensify. Many studies have demonstrated the remarkable relationships between adolescents’ pathological gaming and mental factors, but seldom examined them via longitudinal analysis. With three-year data from adolescents (N = 778) and their parents (N = 685) in South Korea, this study assessed the effects of parents’ negative affect (depression and anxiety) on pathological gaming using adolescents’ mental factors (i.e., aggression, ADHD, self-control). The results showed the critical role of parents’ negative affect on their children’s mental health, which finally leads to pathological gaming among adolescents. Depressive parents increased the degree of adolescents’ aggression and ADHD, and decreased the degree of adolescents’ self-control. Anxious parents increased the degree of adolescents’ ADHD. Moreover, both adolescents’ aggression and self-control mediated the relationship between parents’ depression and adolescents’ pathological gaming. Specifically, self-control was one of the most influential factors contributing to pathological gaming among adolescents.

## 1. Introduction

Parents’ mental health has a strong connection with their children’s psychological upbringing. Parents’ mental problems are associated with children’s poor mental and physical health [1,2]. Specifically, if parents have symptoms of depression and anxiety, their children are highly vulnerable to psychopathological dysfunction, problematic behaviors, or even other serious disorders [3,4].

Although gaming can be a way to achieve relaxation and social connection, excessive use of games (i.e., pathological gaming) could negatively affect adolescents’ mental health and cause conflicts between parents and adolescents [5,6]. Pathological gaming refers to the excessive use of digital games to the extent that one’s social relationships and daily functions are significantly impaired [7]. Specifically, adolescents’ pathological gaming is a worldwide social concern and has attracted increasing research attention in the fields of psychology and cognitive science. Moreover, the negative outcomes of pathological gaming also create conflicts within the family as many of the adolescents still live under their parents’ control [8].

Much has been studied about adolescents’ pathological gaming as one of the most important mental health issues nowadays [9,10,11,12,13,14]. Specifically, according to previous studies, mental health-related factors such as aggression, self-control, and attention-deficit hyperactivity disorder (ADHD) seemed to act as risk factors for pathological gaming [10,15]. It has been reported that aggressive traits showed a strong connection with pathological gaming [16] and pathological involvement with games tends to increase aggression in adolescents [17]. In addition, adolescents with ADHD often lack self-control that puts them at higher risk for pathological gaming [18]. Moreover, self-control is also an important mediator between psychosocial factors and pathological gaming [19]. Few studies have focused on the relationships between adolescents’ pathological gaming and their parents in terms of mental health using longitudinal data.

Moreover, few studies have explored the effects of parents’ depression and anxiety on adolescents’ pathological gaming in line with aggression, ADHD, and self-control. To effectively deal with changes among family relationships as well as adolescents’ pathological gaming after the pandemic, this study used SEM to examine adolescents’ pathological gaming in the context of the parents’ negative affect (depression and anxiety) and their association with adolescents’ aggression, ADHD, and self-control.

## 2. Parents’ Negative Affect and Adolescents’ Mental Health

Anxiety and depressive disorders are common mental illnesses among adults [20]. It is estimated that 4.4% of the global population suffer from depressive disorder, and 3.6% from anxiety disorder [21]. According to the American Psychiatric Association, depression (major depressive disorder) is a common and serious medical illness that causes feelings of sadness and/or a loss of interest in activities, which can lead to a variety of emotional and physical problems [22]. Anxiety (generalized anxiety disorder) encompasses a variety of symptoms, including excessive worrying, restlessness, irritability, difficulties in concentration, and constantly feeling on edge [23].

Parents with depressive and anxiety disorders have risks to transmit their negative affect to their children. Goodman and Gotlib developed a model for understanding the transmission risks for children who have depressive mothers [3]. In their model, they addressed that maternal depression might associate with abnormal development and psychopathology in the children. Aktar and Bögels extended the above model to an integrated one that focuses not only on depression but also anxiety as important negative affect of parents [4]. Their model mainly proposed that early and repeated exposure to parents’ depressive and anxious affect has potential risks on children’s emotional development, and to later psychological outcomes.

According to the integrated model, depressive parents often experience negative affect such as sadness, anger, guilt, etc., that may cause children to acquire negative behaviors and moods that resemble those of their depressed parents. Moreover, the parents’ depressed affect can cause emotional harm to their children, for example, repulsive attitudes might lead to few parent–child interactions. Depressive parents also have more severe emotional changes than non-depressive parents, which might be expressed as an inconsistent parenting style. Thus, the children are not able to develop self-control because of inconsistent parenting behaviors [24]. Anxious parents commonly overreact to specific situations or stimuli, specifically experiencing excessive fear, anxiety, and worry. Anxious parents are not able to provide a positive parent–child interaction due to emotional immaturity. They usually show uncertain feelings about themselves and their children, reflecting a critical attitude toward their children, and they tend to over-control their children [25]. The lack of an optimal interpersonal environment will adversely affect children’s socio-emotional development.

## 3. Pathological Gaming and Adolescents’ Mental Health

Online games have become a leading leisure activity among adolescents. Pathological gaming among adolescents is recognized as a global mental health issue [7]. Pathological gaming is defined as the lack of control in excessive use of games in spite of physical or psychosocial problems [7,9]. In recent years, faced with school closures and pandemic-related stress, there has been a significant rise in the use of games by adolescents. Greater severity of pathological gaming as well as some other related mental problems have also been shown [8].

Mental health factors have a significant impact on adolescents’ pathological gaming [6,26]. Numerous studies have reported mental-related comorbidities in adolescents with pathological gaming, such as aggression [16,17], lack of self-control [5,16], major mental disorders such as depression and anxiety [10,27,28], ADHD [18,29,30], and so on. In particular, previous studies have indicated that time spent on violent games specifically might be associated with aggressive behavior [17,31]. Additionally, self-control has been repeatedly asserted to have a strong relationship with pathological gaming [16]. Some research results showed that the lower the self-control in adolescents is, the higher their dependence and possibilities to have problems in using games become [7,32]. Low self-control means the adolescent probably fails to resist the temptation of the Internet and use of the Internet becomes uncontrollable, so there is a need to guard against the potential of excessive use [33]. For gamers with ADHD who usually seek stimulation, Internet games could continuously offer strong and new stimuli, so adolescents with ADHD are at high risk of falling into pathological gaming [30,34]. ADHD and some psychological factors were found to be the strongest factors associated with pathological gaming in adolescents [30].

### 3.1. Aggression and Pathological Gaming

Aggression is defined as “any behavior intended to harm a target who is motivated to avoid that harm” [31]. According to previous studies, it was found that aggression is related to pathological gaming [17]. In addition, there are a number of studies reporting the association between a preference for violent games and excessive game use [35,36,37]. As reported by a previous study, male players who used games excessively preferred violent video games [17]. To explain the relationship between aggression and excessive game use, searching for a specific type of game is an active process rather than a passive one, and players will select a specific genre of game according to their original tendencies [38]. Another possible reason for aggressive behaviors in gaming is gaming motivation. For example, a player’s aggressive behavior in a game will be repeated as they receive rewards, and this tendency can lead to pathological gaming [16].

### 3.2. Adolescents’ Self-Control and Pathological Gaming

Self-control is the capacity to control or regulate one’s emotion, cognition, and behaviors, and is central to competence [39]. Past studies of self-control and pathological gaming have shown mixed results. Some studies have revealed that self-control-related factors (e.g., impulse control, self-regulation) are negatively related to gaming use [19,40]. Other studies have also demonstrated that self-control accounted for an indirect role between culture [41] or parental behavior and adolescents’ Internet addiction [33].

### 3.3. Adolescents’ ADHD and Pathological Gaming

Attention-deficit hyperactivity disorder (ADHD) is the most common mental disorder among school-aged children. ADHD is defined as a series of behaviors clinically characterized by attention-deficit, hyperactivity, and impulsivity [42]. A previous study suggested that excessive gaming use has a strong connection with symptoms of ADHD and some mental problems such as anxiety, depression, social phobia/anxiety, and obsessive-compulsive symptoms [28]. Individuals with ADHD often lack self-control, putting them at risk for substance abuse and pathological gaming [18]. School-aged children with ADHD show inattention, hyperactivity, and impulsivity, and lack social skills compared to their peers. On the other hand, children with ADHD suffer from many problems due to a lack of attention and impulsive and aggressive behavior. Children with ADHD consistently show emotional problems such as depression and anxiety problems in relationships with parents, and conduct disorder or delinquency [18,43,44].

## 4. Research Model and Hypotheses

To identify the effect of parents’ depression and anxiety on their children’s pathological gaming in line with mental-related factors, this study explored the effects of parents’ depression and anxiety on adolescents’ pathological gaming in line with aggression, ADHD, and self-control.

**Figure 1:**
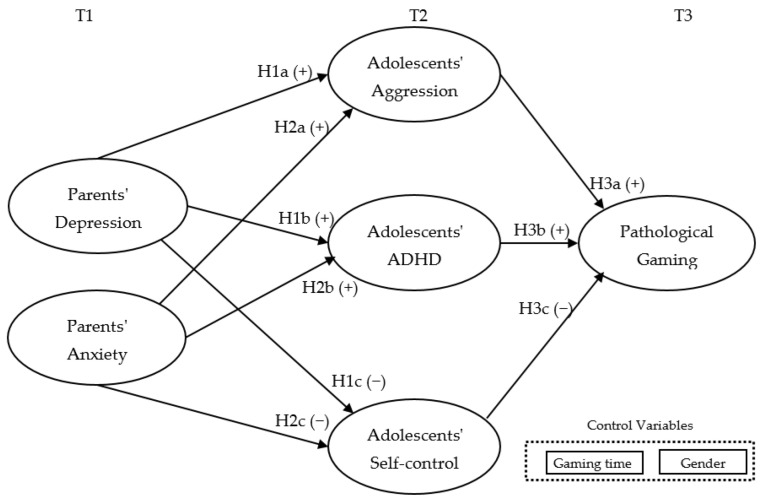
Research model.

The research model (see Figure 1) shows that parents’ negative affect (depression, anxiety) and adolescents’ mental constructs (such as aggression, self-control, ADHD) may influence pathological gaming. Parents’ negative affect can be considered as a contributing factor to adolescents’ mental problems (i.e., high aggression, low self-control, ADHD) that can eventually lead to pathological gaming. Along with these mental-related factors, this study considered the adolescents’ gender and average gaming time per day as control variables to find out if these factors influence pathological gaming.

1. H1. Parents’ depression is positively associated with adolescents’ aggression (H1a) and adolescents’ ADHD (H1b), while parents’ depression is negatively associated with adolescents’ self-control (H1c).
2. H2. Parents’ anxiety is positively associated with adolescents’ aggression (H2a) and adolescents’ ADHD (H2b), while parents’ anxiety is negatively associated with adolescents’ self-control (H2c).
3. H3. Adolescents’ aggression (H3a) and ADHD (H3b) is positively associated with pathological gaming, while adolescents’ self-control is negatively associated with pathological gaming (H3c).
4. H4. Adolescents’ aggression (H4a) and self-control (H4b) mediate the effect of parents’ depression on the degree of pathological gaming.

## 5. Method

### 5.1. Data Collection

This study used the three-year longitudinal data from the Korean Adolescent Game User Cohort Research. The Korea Creative Content Agency (KOCCA) conducted a longitudinal survey to investigate the gaming patterns of elementary, middle, and high school students (ages 10 to 17), while their parents’ psychosocial characteristics were also investigated as important factors that might influence adolescents. Responses were collected 6 times (6-month intervals between each wave) in a face-to-face interview by professional agents, who followed established survey guidelines. Respondents were given the same questionnaire throughout the entire wave. Each participant received USD 27.00 as a reward. The full description of the survey method and data is available on the website (www.kocca.kr, accessed on 25 June 2021).

A total of 778 adolescents and 685 parents (mother 92.4%, other 7.6%) were surveyed for our analysis. Of the total number of adolescents, 381 (49.0%) were boys and 397 (51.0%) were girls. Adolescents were asked about their gaming behaviors and psychological characteristics. Parents were also asked about gaming behaviors as well as psychosocial characteristics.

### 5.2. Measurement

#### 5.2.1. Parents’ Depression

CESD-11 was used to measure parents’ depression. The Center for Epidemiological Studies—Depression Scale (CESD, 20 items) is a self-diagnostic scale for depression developed by the National Institute of Mental Health and used for adolescents and adults. In the “Korea Welfare Panel” survey, CESD-20 was abbreviated to 11 questions. A 4-point scale was used, ranging from “0 = very rare (less than 1 day a week)” to “3 = most of the time (more than 6 days a week)”. It is composed of items such as “I feel depressed,” “I feel lonely,” and “I feel others dislike me.”

#### 5.2.2. Parents’ Anxiety

To measure parents’ anxiety, the generalized anxiety disorder (GAD) scale developed by Spitzer, Kroenke, Williams, and Löwe [45] was used. It consists of a total of 6 items and was rated on a 4-point scale ranging from “0 = not at all” to “3 = mostly.” It is composed of items such as “I was very worried about various things,” “I couldn’t control my worries,” and “I was anxious about things not going to happen or what would happen.”

#### 5.2.3. Adolescents’ Aggression

The Short-Form Buss–Perry Aggression Questionnaire (BPAQ-SF) was used to measure adolescent aggression. Bryant and Smith [46] reduced the 29-item aggression scale, BPAQ (The Buss–Perry Aggression Questionnaire) devised by Buss and Perry [47], to 12 items. They were rated on a 5-point scale from “1 = not at all” to “5 = very much.”

#### 5.2.4. Adolescents’ Self-Control

To measure adolescents’ self-control, the Brief Self-Control Scale (BSCS), a scale developed by Tangney and his colleagues [48], was used, and consisted of a total of 13 items rated on a 5-point scale, ranging from “1 = not at all” to “5 = very much.” It consisted of items such as “I don’t give in to temptation,” “Sometimes I get so caught up in the fun that I don’t get the job done,” and “I often act without thinking about other solutions.”

#### 5.2.5. Adolescents’ ADHD

ADHD in adolescents was measured using parent-reported questionnaire data. To this end, a questionnaire for rating by parents was used from the Korean ADHD Rating Scale (K-ARS), which was developed by DuPaul [44] based on the ADHD diagnostic criteria of DSM-IV. A total of 18 items were rated on a 4-point scale, from “0 = not at all” to “3 = strongly agree,” by checking the number that best describes the behavior of the child during the week. It was composed of items such as “I can’t stay and keep moving my hands and feet or wriggling,” “I have difficulty doing tasks or activities systematically,” and “I can’t wait for my turn.”

#### 5.2.6. Adolescents’ Pathological Gaming

To measure the degree of pathological gaming in adolescents, a modified version of the existing Internet Addiction Scale [49] was used to suit the game situation. It consisted of a total of 20 items and was rated on a 5-point scale ranging from “1 = not at all” to “5 = very much.” The questionnaire consists of items such as “I neglect other things because of the game,” “I have problems in school because of the game,” and “I feel bored and empty without the game.”

#### 5.2.7. Daily Gaming Time

For the daily gaming time, the average daily gaming time was asked as follows: “How many hours do you play games a day?” with 9 response options: 1 (None), 2 (<30 min), 3 (30 min–1 h), 4 (1–2 h), 5 (2–3 h), 6 (3–4 h), 7 (4–5 h), 8 (5–6 h), and 9 (over 6 h).

## 6. Results

### 6.1. Reliability and Validity Test

The levels of depression and anxiety of 685 parents (T1) and ADHD of adolescents (T2) reported by parents, and the degree of aggression (T2), self-control (T2), and pathological gaming (T3) of 778 adolescents in South Korea were measured.

Reliability and correlations as well as validity tests were performed for the measurement values obtained (see Table 1 and Table 2). The reliability test results included Cronbach’s alpha, composite reliability (CR), and average variance extracted (AVE). The resulting scores were all valid for the model test (0.8 for CR and 0.5 for AVE). For missing data, a regression imputation method in the Amos program was used. This method replaces missing data with substituted values by making linear regressions among variables.

### 6.2. Research Model Test

For the research questions, structural equation analysis was conducted using Amos 22.0. The results yielded valid and adequate indices for the model fit: IFI = 0.947, CFI = 0.947, and RMSEA = 0.047.

**Figure 2:**
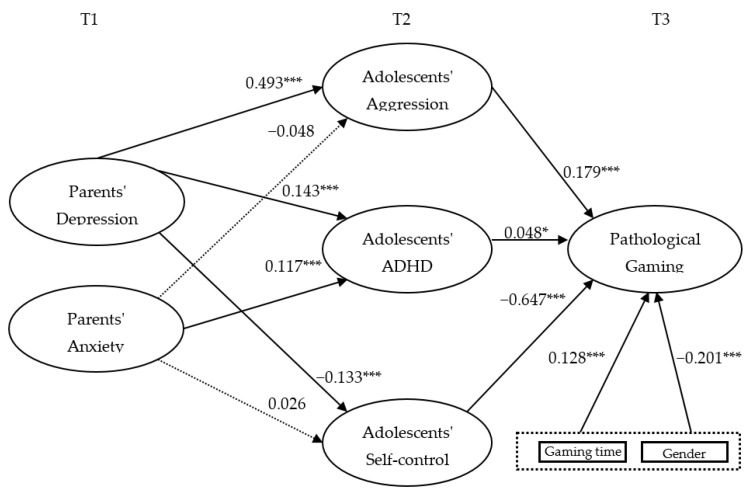
Research model and hypothesis. * *p* < 0.01, *** *p* < 0.0001.

The results of hypothesis tests showed that all hypothesis are accepted except H2a and H2c (see Table 3). The results presented in Figure 2 showed that parents’ depression in T1 had a positive effect on aggression (0.493) and ADHD (0.143), and a negative effect on self-control (−0.133) of T2 children. Parents’ anxiety in T1 had a positive effect on ADHD (0.117) in children of T2 and had no significant effect on aggression and self-control. Child aggression, ADHD, and self-control in T2 all had a significant effect on pathological gaming in T3. Specifically, aggression (0.179) and ADHD (0.048) had a positive effect on pathological gaming, while self-control (−0.647) had a negative effect. The control variables, gender (−0.201) and T2’s gaming time (0.128), also had a significant effect on T3’s pathological gaming.

### 6.3. Mediation Test (Parents’ Depression–Adolescents’ Aggression, Self-Control–Pathological Gaming)

To determine the mediating effect of adolescents’ aggression and self-control, four conditions were tested: (1) whether parents’ depression affected adolescents’ aggression, (2) whether adolescents’ aggression affected pathological gaming, (3) whether parents’ depression affected adolescents’ self-control, and (4) whether adolescents’ self-control affected pathological gaming. The results showed that parents’ depression significantly affected adolescents’ aggression (β = 0.774, *p* < 0.001), while adolescents’ aggression showed a significant effect on pathological gaming (β = 0.420, *p* < 0.001). Parents’ depression also significantly affected adolescents’ self-control (β = −0.742, *p* < 0.001), and adolescents’ self-control showed a significant effect on pathological gaming (β = −0.243, *p* < 0.001).

To confirm the mediating effect of adolescents’ aggression and self-control, Sobel’s test was conducted. The bootstrapped 95% confidence intervals were checked for the standardized indirect effect. The mediated effect of parents’ depression through adolescents’ aggression was significant (Sobel’s z = 8.152, *p* < 0.001; 95% CI = (0.26, 0.40)). The mediated effect of parents’ depression through adolescents’ self-control was also significant (Sobel’s z = 3.317, *p* < 0.001; 95% CI = (0.26, 0.40)). Thus, both adolescents’ aggression and self-control were confirmed to mediate the effect of parents’ depression on the degree of pathological gaming.

## 7. Discussion

### 7.1. Findings

This study investigated the effect of parents’ negative affect on children’s mental health by analyzing longitudinal data. The research model focused on the influence of depressive and anxious parents on their children’s pathological gaming through aggression, self-control, and ADHD. By analyzing SEM, this study found some important findings.

Firstly, depressive parents played a key role in adolescents becoming pathological gamers. Specifically, parents’ depression increased children’s aggression, ADHD, and decreased self-control, which ultimately led to pathological gaming. Adolescents’ aggression and ADHD increased, while self-control decreased pathological gaming. Parents’ anxiety increased their children’s ADHD, however it did not affect aggression and self-control.

Secondly, this study found that adolescents’ self-control and aggression mediated the relationship between pathological gaming and parents’ depression. It suggested that adolescents whose parents have a high degree of depression are much more vulnerable to pathological gaming if they have a low degree of self-control or a high degree of aggression. Adolescents with low self-control whose parents have a high degree of depression are more at risk of engaging in pathological gaming. Adolescents with a high level of aggression whose parents have a high degree of depression also have a higher risk to engage in pathological gaming. In other words, when parents have a high degree of depression, adolescents become much more susceptible to pathological gaming through two different paths: low self-control or high aggression. However, adolescents’ self-control and aggression can play an important role as preventive factors for pathological gaming. Even if parents have a high degree of depression, if adolescents’ self-control improved or their aggression is reduced, then adolescents would also be able to reduce their pathological gaming tendencies.

Thirdly, it was found that self-control was one of the most influential factors contributing to adolescents’ pathological gaming. Factors such as gaming time, self-control, aggression, and ADHD all led to pathological gaming after one year. However, self-control showed a much greater influence on pathological gaming than other factors. In particular, lower self-control led to a higher risk of pathological gaming than gaming time, and this result supports the previous research [7]. Adolescents with higher self-control had a lower risk of pathological gaming [5] regardless of gaming time. These results suggest that self-control is more influential than gaming time as a direct contributing factor to adolescents’ pathological gaming.

### 7.2. Theoretical and Practical Implications

This study investigated the effect of parents’ negative affect on children’s mental health by analyzing longitudinal data. The research model focused on the influence of depressive and anxious parents on their children’s pathological gaming through aggression, self-control, and ADHD. This study’s theoretical and practical implications are as follows. There have been few studies that investigated adolescents’ pathological gaming from the perspective of parents’ depression and anxiety through their children’s mental health. As far as we know, this is the first attempt. This study also investigated the relationship using three-year longitudinal data. Although the number of longitudinal studies examining adolescents’ pathological gaming is increasing [50,51,52], only a few studies focus on both parents’ negative affect and their children’s mental health-related factors. Remarkably, this study found two mechanisms by which parents’ depression decreased adolescents’ self-control/increased aggression, and both could lead to pathological gaming. Finally, this study was able to present much more accurate research results than previous studies because data were collected from both parents and children through self-reporting.

The present study also provided guidance for ways to prevent adolescents’ pathological gaming. Governments and institutions might consider more of a focus on parents’ negative affect and adolescents’ mental health. For example, there should be more help for parents to control their depression and anxiety. As for adolescents, there should be a focus on mental health management, especially strengthening self-control and reducing aggression. Along with control of gaming time, a positive family environment, where adolescents form their characteristics, is important in establishing and promoting the adolescents’ self-control as well as reducing their aggression and pathological gaming tendencies.

Although this research mainly focused on the relationships among parents’ negative affect (depression and anxiety) and their children’s mental health-related factors as well as pathological gaming, there exist some other important psychosocial variables and environmental factors. Thus, future studies may consider the mental health of adolescents and their parents more as well as the quality of relationship with peers and teachers to increase the explanatory power of adolescents’ pathological gaming.

Another limitation of this study has to do with sampling. This study merely analyzed data of parents and adolescents from South Korea, while different results may be generated from different countries. Thus, future studies need to collect data from various countries to compare the differences or to facilitate generalization of the results.

The correlation between self-control and ADHD was non-significant. Possible explanations are that the sample of adolescents had normal levels of ADHD with low severity. The correlation also had a small standard deviation. Therefore, the correlation between self-control and ADHD may seem non-existent. A limitation of this study is that we were not able to study adolescents with varying degrees of ADHD, and therefore future research will be needed.

The purpose of this study was to survey general households in South Korea, but deviations in gender ratios was a limitation of this study. The survey was conducted during the daytime. However, in South Korea, it is common that the father goes to work during the day, and the mother takes care of the housework, so it can be interpreted that this was why the majority of the survey subjects were mothers.

This study did not take a medical approach to factors such as parents’ anxiety and depression, their children’s ADHD, and pathological gaming. This paper studied the difference in influence that exists between each factor. However, in-depth analysis of each variable needs to be continuously studied in the future.

## 8. Conclusions

In summary, this study identified the effects of parents’ depression and anxiety as important mental health-related factors contributing to pathological gaming among adolescents. The degree by which adolescents’ aggression and self-control increased/decreased in relation to the parents’ depression constitutes one of the most important results of this study. It is suggested that improving self-control and reducing aggression can be vital in preventing pathological gaming among adolescents, but it is also important that parents can learn to control their depressive affect.

## Figures and Tables

**Table 1:** Reliability and discriminant validity of constructs.

Constructs	No. of Measures	Mean (SD)	Cronbach’s Alpha	CR	AVE
Depression (P-t1 ^1^)	6	1.45 (2.359)	0.835	0.861	0.510
Anxiety (P-t1 ^1^)	6	3.60 (3.145)	0.887	0.892	0.579
Aggression (A-t2 ^2^)	5	9.17 (4.101)	0.872	0.887	0.567
ADHD (A-t2 ^2^)	6	3.00 (2.974)	0.867	0.869	0.527
Self-control (A-t2 ^2^)	9	41.48 (22.714)	0.991	0.991	0.930
Pathological gaming (A-t3 ^3^)	19	41.82 (16.523)	0.954	0.977	0.690

^1^ P-t1: Time 1 of parents. ^2^ A-t2: Time 2 of adolescents. ^3^ A-t3: Time 3 of adolescents.

**Table 2:** Correlations and discriminant validity analysis.

Constructs	1	2	3	4	5	6
Depression (P-t1)	**0.714**					
Anxiety (P-t1)	0.571 ***	**0.761**				
Aggression (A-t2)	0.465 ***	0.185 ***	**0.753**			
ADHD (A-t2)	0.189 ***	0.186 ***	0.229 ***	**0.726**		
Self-control (A-t2)	0.111 **	−0.041	−0.148 ***	0.008	**0.964**	
Pathological gaming (A-t3)	0.215 ***	0.099 **	0.304 ***	0.115 ***	−0.657 ***	**0.831**

The square root of AVE (average variance extracted) is presented in boldface in the diagonal cells for the corresponding construct. ** *p* < 0.001, *** *p* < 0.0001.

**Table 3:** Results of the hypothesis tests.

Hypothesis	B	β	SE	CR	Results
H1a. Parents’ depression → Adolescents’ aggression	0.873	0.493	0.070	12.540	Accepted
H1b. Parents’ depression → Adolescents’ ADHD	0.145	0.143	0.037	3.955	Accepted
H1c. Parents’ depression → Adolescents’ self-control	−0.952	−0.133	0.242	−3.929	Accepted
H2a. Parents’ anxiety → Adolescents’ aggression	−0.068	−0.048	0.045	−1.513	Rejected
H2b. Parents’ anxiety → Adolescents’ ADHD	0.094	0.117	0.029	3.296	Accepted
H2c. Parents’ anxiety → Adolescents’ self-control	0.148	0.026	0.188	0.788	Rejected
H3a. Adolescents’ aggression → Pathological gaming	0.252	0.179	0.035	7.207	Accepted
H3b. Adolescents’ ADHD → Pathological gaming	0.118	0.048	0.059	2.009	Accepted
H3c. Adolescents’ self-control → Pathological gaming	−0.224	−0.647	0.009	−23.661	Accepted

## Data Availability

The data used in this study are available with permission from the Korea Creative Content Agency (KOCCA, http://www.kocca.kr, accessed on 20 May 2021).
